# Herramientas de sistemas de información geográfica para delimitar áreas en centros de salud familiar chilenos. Conceptos desde la práctica

**DOI:** 10.15446/rsap.V26n5.116376

**Published:** 2024-09-01

**Authors:** Sandra Soto-Provoste, Angela Rebolledo-Poblete, Greys González-González

**Affiliations:** 1 SS: Sociól. M. Sc. Salud Pública. Universidad de Chile. Departamento de Salud Municipal, Municipalidad de Temuco. Temuco, Chile. sotoprovoste@gmail.com Departamento de Salud Municipal Municipalidad de Temuco Temuco Chile; 2 AR: Ing. Geomesora. Secretaría Comunal de Planificación, Municipalidad de Temuco. Temuco, Chile. angela.rebolledo@temuco.cl Municipalidad de Temuco Temuco Chile angela.rebolledo@temuco.cl; 3 GG: Enf. M Sc. Enfermería. Mención Gestión del Cuidado, Universidad de la Frontera. Departamento de Enfermería, Universidad de La Frontera. Temuco, Chile. greys.gonzalez@ufrontera.cl Universidad de La Frontera Departamento de Enfermería Universidad de La Frontera Temuco Chile

**Keywords:** Sistemas de información geográfica, salud pública, salud pública digital *(fuente: DeCS, BIREME)*, Geographic information systems, public health, public health informatics *(source: MeSH, NLM)*

## Abstract

El uso del sistema de información geográfica (SIG) en la planificación de la salud ha demostrado ser fundamental, especialmente al considerar factores como la movilidad, el transporte y el crecimiento urbano. El crecimiento urbano sin regulación tiende a concentrar y segregar los servicios, dejando a las poblaciones más necesitadas, en la periferia sin acceso adecuado a la atención sanitaria (AS). El análisis de información geoespacial y su integración en la planificación sanitaria no solo mejora la distribución de los servicios, sino que facilita la toma de decisiones basada en una comprensión más completa del territorio. La implementación de estrategias SIG puede contribuir a una mejor equidad en la AS y a una planificación urbana más inclusiva y eficaz. Se presenta un ensayo con reflexiones sobre el uso de SIG en una comuna del sur de Chile.

## Abstract (en)

The use of geographic information systems (SIG) in health planning has proven to be essential, especially when considering factors such as mobility, transportation, and urban growth. Unregulated urban growth tends to concentrate and segregate services, leaving the most needy populations on the periphery without adequate access to healthcare. The analysis of geospatial information and its integration into health planning not only improves the distribution of services but also facilitates decision-making based on a more comprehensive understanding of the territory. Implementing GIS strategies can contribute to better equity in healthcare and more inclusive and effective urban planning. An essay presents reflections on using GIS in a city of southern Chile.

Las herramientas de información geográfica en salud han sido ampliamente utilizadas y han evolucionado paralelamente al cambio en la mirada de los conceptos de territorio y salud. Dichos conceptos se han ido complejizando, y en la actualidad son más transdisciplinarios e integradores; se considera que el territorio es más que lo geográfico y salud es más que un concepto biomédico 1.

La toma de decisiones en la salud pública requiere y necesita información con componente territorial, ya sea para determinar un brote o para distribuir los recursos 2. Los sistemas de información geográfica (SIG) se consideran herramientas cuantitativas esenciales para analizar información espacial desde múltiples fuentes 3, utilizando un enfoque geoespacial y cualitativo emergente para comprender de manera integral los problemas de salud. Los SIG permiten consultar de manera interactiva información geográfica digital (latitud, longitud, altitud), facilitando con ello la combinación e integración de múltiples cartografías, manejadas como capas superpuestas de datos digitales que se pueden observar de manera simultánea 4,5.

Se recomienda e incentiva realizar investigaciones sobre los SIG, ya que, a través de la utilización de métodos y aplicaciones de datos espaciales sólidos, con fuentes de datos seguras y confiables se puede generar información sobre el impacto de las características sociodemográficas de la población en su situación de salud 6. Según la propuesta política geoespacial de Chile del año 2023 7, los sistemas SIG entregan información de manera confiable, actualizada y oportuna, que es a su vez capaz de apoyar la toma de decisiones basadas en la evidencia. La información geoespacial es un insumo básico para la gestión y la planificación que se realiza en las instituciones del Estado, por lo que requiere orden, estandarización y ser sistematizada para su uso.

La división político-administrativa del territorio chileno subdivide al país en regiones, provincias y comunas, siendo esta última, administrada por un municipio, la desagregación básica de la administración pública 7,8. En el ámbito de la salud, son principalmente los centros de salud familiar (Cesfam), además de los centros de salud comunitarios y familiares (Cecosf) y las postas, dependientes del municipio, los que se encargan de administrar la oferta sanitaria en el territorio, para lo cual hacen un trabajo de sectorialización del espacio donde se encuentran, además de un mapeo epidemiológico de su población a cargo, lo cual se puede apreciar en los mapas físicos colgados de alguna pared en todos los Cesfam con algún marcador que identifique sectores y alfileres con la población a cargo 8. Como hasta la fecha esta labor no ha sido planificada ni abordada por la comuna en su conjunto, la incorporación de poblaciones, sectores y villas a la labor de los Cesfam ha generado lugares dentro de la comuna sin cobertura, o en el caso de los más densamente poblados no se tienen claros los límites territoriales de uno u otro Cesfam 8. Debido a esto, es fundamental incorporar nuevos elementos a la visión del territorio, siendo posible la utilización de herramientas SIG, incorporando elementos como áreas verdes y determinantes sociales, lo que facilita la coordinación entre los programas de atención médica que tienen los centros de salud 9.

En Chile, la atención primaria de salud basa su quehacer en el Modelo de Atención Integral en Salud Familiar y Comunitaria (MAIS), que tiene sustento en tres principios: centralidad en las personas, integralidad de la atención y continuidad de los cuidados. Para trabajar en relación con estos tres pilares, el MAIS necesita un trabajo intersectorial que requiere personal sanitario que cuente con una visión completa y acabada del territorio 8,9. La atención primaria de salud se organiza en los Cesfam, además de los Cecosf y las postas rurales 10. Los Cesfam son establecimientos de atención primaria que atienden a poblaciones asignadas de entre 5 000 y 30 000 inscritos validados dentro de una comuna y constituyen el primer nivel de complejidad en la atención de salud 10.

El presente ensayo presenta una estrategia de estudio de delimitación de áreas de influencia de los Cesfam de la comuna de Temuco, región de la Araucanía, Chile, mediante el uso de SIG, abordando la importancia de esta herramienta y generando debate sobre su manejo y eficiencia en la planificación sanitaria de equipos de atención primaria de salud.

## Generalidades de la población y descripción del modelo

La información que se entrega a continuación fue obtenida del Plan de Desarrollo Comunal (Pladeco) 11. Temuco es la capital de la región de la Araucanía y de la provincia de Cautín. Se ubica a 667 km al sur de Santiago. La superficie total de la comuna es de 464 km2, de la cual el 71% corresponde a la zona rural y el 29% es urbana.

Según las proyecciones del Instituto Nacional de Estadísticas (INE), con base en el censo de 2017, la población estimada al 2024 para la comuna de Temuco es de 309 696 habitantes, de los cuales el 93% reside en el área urbana. De acuerdo con la previsión de salud, un 80% es beneficiario de Fondo Nacional de Salud (Fonasa).

El municipio de Temuco, por medio del Departamento de Salud Municipal (DSM), administra y coordina el plan de salud, que orienta los recursos humanos, materiales y financieros para el desarrollo de estrategias y políticas de salud local. Estas últimas se ejecutan de acuerdo con el Modelo de Atención de Salud Familiar, a través de la red de establecimientos de salud de la comuna, la cual se compone de ocho Cesfam y tres Centros Comunitarios de Salud Municipal (Cecosf).

El Departamento de Salud Municipal de Temuco se financia principalmente mediante los aportes de la Ley N.° 19.378, basados en el número de beneficiarios inscritos en los establecimientos de salud, y el mecanismo de financia-miento per cápita, que cubre el 63% del presupuesto total del área de salud para el año 2024 en la comuna 12.

La estructura de inscritos en los centros de salud de Temuco presenta un perfil predominantemente femenino, con un 53% de mujeres, y se concentra en la franja etaria de 15 a 34 años, que corresponde a la edad productiva. Al analizar la distribución por grupos de edad, se observa que las proporciones más bajas corresponden a los extremos etarios, destacando especialmente la baja inscripción de personas mayores de 65 años.

El MAIS establece que el conocimiento de la población a cargo es fundamental para implementar un enfoque biopsicosocial y garantizar la continuidad de los cuidados. Una de las estrategias para acercarse a la población usuaria es la sectorialización, que implica dividir el territorio en áreas específicas, considerando barreras geográficas y límites naturales.

La sectorialización se basa en varios elementos clave: una superficie determinada, un número específico de habitantes, la identificación de equipamiento e infraestructura comunitaria y la organización de recursos. Esta estrategia busca facilitar un ordenamiento territorial que permita una atención más efectiva y cercana a las necesidades de la población. En resumen, la sectorialización es una herramienta que contribuye a mejorar la atención primaria, al organizar y optimizar los recursos disponibles en función de la población asignada.

Un sector se define como un espacio territorial donde reside un grupo poblacional que forma la "población a cargo" de un equipo de salud. La sectorialización implica dividir este territorio considerando barreras geográficas y límites naturales, reconociendo elementos como la superficie, el número de habitantes y la identificación de la infraestructura comunitaria. Esta estrategia se orienta a facilitar el acercamiento a la población usuaria y optimizar la organización de los recursos en salud. Como criterio de sectorialización se plantea dividir la población total en función del número estimado de usuarios por sector, lo que permitirá analizar su relación con las poblaciones y los límites de las juntas de vecinos, así como los factores de riesgo asociados. Además, se sugiere segmentar la población según el número de familias inscritas y considerar variables geográfico-administrativas que faciliten la implementación de acciones sanitarias operativas. Esto incluye la evaluación de variables de riesgo biopsicosocial y la disponibilidad de recursos humanos. Es fundamental respetar las áreas geográficas de las juntas de vecinos, ya que son actores clave en el proceso de participación y colaboración en el trabajo de salud.

## Delimitación de áreas de influencia, mecanismos SIG

El trabajo de delimitación de áreas se llevó a cabo utilizando la metodología de cartografía social, también conocida como mapa social o sociograma. Esta técnica permite llevar a cabo una aproximación a un territorio específico, fomentando un sentido de pertenencia a través de representaciones gráficas de la realidad comunitaria. Se entiende el territorio como un espacio dinámico y en constante evolución.

Las actividades que se llevaron a cabo fueron:

- Revisión bibliográfica: de orientaciones técnicas ministeriales, planes de desarrollo municipal, entre otros, donde se aborde el tema de la sectorialización o las áreas de influencia.
- Recopilación de mapas de sectorización de los Cesfam, físicos o en formato digital.
- Reuniones con los equipos de los Cesfam de la comuna de Temuco.
- Definición conjunta y jerarquización de criterios de delimitación.
- Subir polígonos a visualizador Web Mapping Application.
- Descargar información de población inscrita validada de los Cesfam desde Rayen.
- Georreferenciación de domicilios.
- Generar capas de áreas de influencia y de población inscrita.
- Concordar escenarios de trabajo sanitario en la comuna.

Para realizar esto se llevaron a cabo reuniones con los equipos de cada establecimiento, en las que participaron personas con conocimiento y trabajo en el territorio como directores/as, subdirectores/as médicos/as, encargados/ as de visitas domiciliarias (PADI), participación ciudadana y gestores de información (GDI). En estas reuniones, los equipos identificaron los criterios y los aspectos relevantes para delimitar sus áreas de intervención. Los conceptos de delimitación fueron afinándose en cuanto a conceptualización y jerarquía. Posteriormente, la información obtenida se contrastó en reuniones con los directivos de todos los establecimientos de atención primaria de la comuna, con el fin de darles una mirada comunal y acordar el funcionamiento en los límites compartidos y áreas sin establecimiento a cargo. Tras definir los polígonos con los equipos, se dibujaron las áreas de influencia y se levantaron en un visor de acceso compartido, donde también se georreferenciaron los establecimientos de atención primaria, hospitales, bomberos, escuelas y otra información relevante del territorio.

Los criterios que emergieron de esto fueron: *universalidad,* el cual buscaba asimilar los polígonos de influencia de los Cesfam a los límites urbanos de la comuna y otros límites territoriales definidos por el municipio, como los macrsectores. Este enfoque permite que, al agregar información georreferenciada, se garantice la coincidencia con los datos de otras áreas que intervienen en el trabajo intersectorial. Además, se consideraron límites naturales, como el cerro Ñielol, ríos y puentes, lo que contribuye a incorporar las áreas verdes y características naturales de la planificación sanitaria del territorio. El segundo fue el *criterio de territorialidad,* el cual se llevó a cabo definiendo en el mapa vial, las calles, las villas y las poblaciones que se incluían en el perímetro de influencia del Cesfam. Asimismo, se incorporó la identidad territorial de los barrios; por ejemplo, juntas de vecinos o asociaciones de barrios. Un tercer criterio fue el de *movilidad,* concepto relacionado con el acceso y la dependencia, entendiendo, por una parte, qué tan factible es llegar a los domicilios para el equipo de salud y, por parte de las personas, qué tan factible es acceder al centro de salud (para población con dependencia severa o problemas de movilidad, por ejemplo), contemplando accesibilidad desde el transporte público, vías de acceso vehicular y peatonal. Por último, se encuentra el criterio *poblacional,* el cual se basó en la geolocalización de los domicilios de la población inscrita en cada Cesfam, para contrastar si la capa poblacional coincidía con lo que habían acordado los equipos.

Los tres primeros criterios fueron definidos por los equipos de los Cesfam y el criterio poblacional se levantó con la información descargada de la base de datos de los establecimientos, mediante el Sistema Iris Rayen, para la gestión de información de pacientes de los establecimientos de atención primaria de salud.

**Figura 1:**
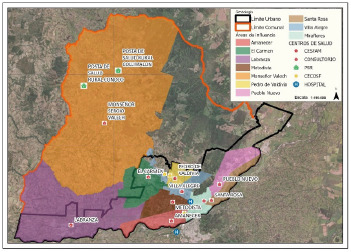
Áreas de influencia de los Cesfam de la comuna de Temuco

La información resultante arrojó las áreas de influencia contenidas en la Figura 1, donde los Cesfam Valech y Labranza contemplan a la población que reside en las áreas rurales de la comuna, mientras que el resto de la población se ubica en el sector urbano.

Fuente: Secretaría Comunal de Planificación (SECPLA), Municipalidad de Temuco.

**Figura 2:**
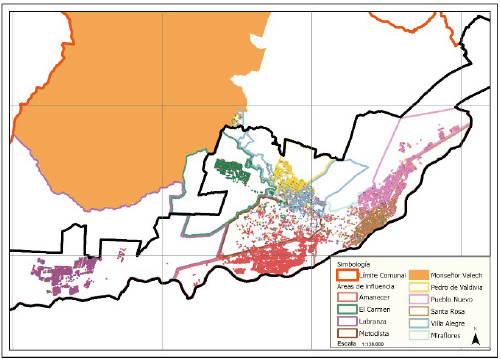
Población inscrit3a según Cesfam en la Comuna de Temuco

Al comparar las áreas de influencia con la georreferenciación de los domicilios de la población inscrita por centro de salud, vemos que la información de la población georreferenciada se encuentra, en su mayoría, dentro del perímetro de influencia de los Cesfam, a excepción de los sectores urbanos densamente poblados, como el límite entre Villa Alegre y El Carmen, Amanecer y el límite entre Pueblo Nuevo y Santa Rosa, como se describe en la (Figura 2).

Fuente: SECPLA, Municipalidad de Temuco.

**Figura 3:**
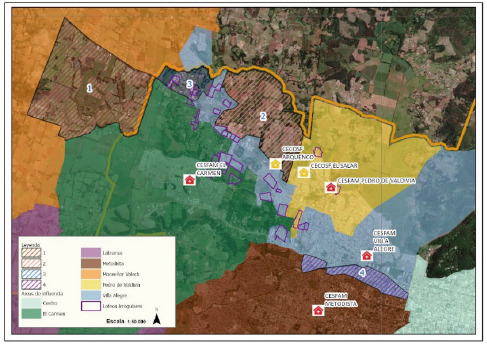
Sectores sin circunscripción de influencia dentro del área urbana de la Comuna de Temuco

Una vez terminada la definición de los polígonos correspondientes a cada Cesfam quedaron dos sectores sin áreas de influencia (Figura 3), los cuales corresponden a sitios dentro del área urbana donde se proyecta inversión inmobiliaria en el futuro, y con viviendas no urbanizadas. Por ello, los Cesfam consideraron que era necesaria la incorporación de otros elementos en el análisis territorial sanitario para adscribir más territorio del que ya administraban.

Fuente: SECPLA, Municipalidad de Temuco.

Por otra parte, queda un sector con doble área de influencia: el sector 4, donde se superponen el Cesfam de Villa Alegre y el Cesfam Metodista; ambos manifestaron tener pacientes y visitas domiciliarias en esa área común. Se optó por dejar esta área con la superposición, ya que el Cesfam Metodista no es de dependencia municipal, sino que es administrado por una congregación religiosa, y además en el sector, que es alta plusvalía, se concentran algunas residencias de adultos mayores, con población que se atiende en el sector privado.

### Consideraciones finales

Desde los romanos, pasando por J. Snow, los mapas han facilitado la comprensión de los fenómenos de salud que afectan a las personas. Con el advenimiento de las nuevas tecnologías, estos mapas han incorporado elementos sociales a un territorio, logrando visibilizar, por ejemplo, los determinantes sociales en salud. Lo que buscamos aquí es explorar su utilidad en la planificación de la atención primaria de salud, que es la primera línea de la política pública en las comunas del país.

La delimitación de áreas de influencia para los Centros de Salud Familiar (Cesfam) en la comuna de Temuco recoge, con ayuda de herramientas tecnológicas, el trabajo de vacunación en domicilio en contextos de pandemia y el trabajo de promoción que realizan los funcionarios de salud día a día en las calles, escuelas, juntas de vecinos de la comuna.

El uso del SIG en la planificación de la salud ha demostrado ser fundamental, especialmente al considerar factores como la movilidad, el transporte y el crecimiento urbano. El crecimiento urbano sin regulación tiende a concentrar y segregar los servicios, dejando a las poblaciones más necesitadas, en la periferia, sin acceso adecuado a la atención sanitaria.

Además, investigaciones han demostrado que la integración de técnicas SIG con análisis de datos demográficos y de movilidad urbana puede mejorar significativamente la planificación sanitaria.

Es crucial también incluir otros actores, como inmobiliarias y organismos de transporte, en la planificación urbana en salud. Esto se debe a que la ubicación de los centros de salud no siempre sigue el crecimiento urbano, lo que puede dejar a comunidades marginadas, como las rurales o las comunidades mapuches con títulos de merced, sin una adecuada atención sanitaria. La colaboración intersectorial y la depuración de bases de datos, así como la validación de la información por Fonasa, son pasos fundamentales para mejorar la precisión y la eficacia de la planificación sanitaria a nivel comunal.

El análisis de información geoespacial y su integración en la planificación sanitaria no solo mejora la distribución de los servicios, sino que facilita la toma de decisiones basada en una comprensión más completa del territorio. La implementación de estrategias SIG puede contribuir a una mejor equidad en la atención sanitaria y a una planificación urbana más inclusiva y eficaz ♦
